# Aggressive and Withdrawn Behaviour at School through the Lens of Teachers and Peers: A Qualitative Study

**DOI:** 10.3390/bs14050412

**Published:** 2024-05-15

**Authors:** Susanna Pallini, Antonia Lonigro, Barbara Barcaccia, Fiorenzo Laghi, Barry H. Schneider

**Affiliations:** 1 Department of Education, Roma Tre University, 00185 Rome, Italy; 2 Department of Psychology, Sapienza University of Rome, 00185 Rome, Italy; 3 Associazione di Psicologia Cognitiva APC and Scuola di Psicoterapia Cognitiva Srl SPC, 00185 Rome, Italy; 4 Department of Social and Developmental Psychology, Sapienza University of Rome, 00185 Rome, Italy; 5 Department of Psychology, Boston College, Boston, MA 02467, USA

**Keywords:** social withdrawal, aggression, teachers, sociometric peer status

## Abstract

We explored teachers’ understanding of children with aggressive or socially withdrawn behaviour in their classes and we associated our findings with a status of rejected, neglected, or popular, as provided by peer nominations. Five kindergarten and elementary school teachers scored their 143 pupils with the Child Behaviour Checklist for Withdrawal and Aggression. Subsequently, only those children whose scores were 1 standard deviation above the mean for withdrawal or for aggression were included in the final sample (*n* = 46; M_age_ = 6.5 years, *SD* = 1.7; age range = 4–9 years). The final sample included 31 children (21.67%; females = 16) who were assessed as displaying withdrawn behaviour, and 15 (10.48%; females = 5) displaying aggressive behaviour. An open-ended semi-structured interview was administered to teachers, who described children with socially withdrawn behaviour as introverted and untalkative, children with aggressive behaviour as hostile, rule-breaking and highly active, and children with socially withdrawn or aggressive behaviour as isolated, even though different reasons were provided. The results of the sociometric status in children with socially withdrawn or aggressive behaviour are discussed.

## 1. Introduction

In the last few years in Italy, as in many other countries in the Organization for Economic Co-operation and Development (OECD), an educational reform process has been taking place aimed at reducing social exclusion in the classroom [1], that is “the experience of being kept apart from others physically or emotionally” [2]. Specifically, social exclusion may regard both children exhibiting aggressive behaviours (creating a management problem in school settings and impairing learning processes [3,4]) and children withdrawing into themselves (who have consequent interpersonal difficulties with their classmates). Cross-sectional and longitudinal studies have confirmed a strong relation between peer rejection and overt or relational forms of aggressive behaviours in childhood and adolescence (for a meta-analysis, see [5]) as well as between peer rejection and withdrawn behaviour (for a commentary, see [6]).

According to Achenbach [7] children’s aggressive behaviours encompass fighting, attacks, screaming, threats, destruction, or being stubborn, whereas withdrawn behaviours encompass the preference to be alone, the tendency to not talk, and shyness. In the School Children Mental Health Europe (SCMHE) study, a cross-sectional survey of European children aged 6–11, the authors compared the prevalence of children’s psychological problems across Europe [8]. They found that among Italian children, the percentage of teachers who reported very high scores of conduct problems (including aggressive behaviour) was 4.9%, and of peer problems (including withdrawn behaviour) was 4.4%, whereas the European range was between 3.5% and 6% for conduct problems, and between 3.3% and 6.8% for peer problems; thus, this is a common and notable issue in the Italian context.

### 1.1. Aggressive/Withdrawn Behaviours and Interpersonal Problems

Children with aggressive or socially withdrawn behaviour may have interpersonal yet opposite problems, and both are at risk for social maladjustment [9,10]. The complete or partial lack of early identification of children’s behavioural problems may constitute a lost chance for planning suitable intervention programs, accruing problems in social interactions among classmates [11].

On the one hand, some children with aggressive behaviour may show impulsive reactions to stressful events; in frustrating situations, they may show emotional reactions such as anger and hostility, and in peer conflict situations, they may overreact. Other children with aggressive behaviour may think that their behaviour is normal, without reflecting on the sadness and pain that they elicit in others [12,13,14].

On the other hand, social withdrawal encompasses different aspects such as the lack of interest in peer engagement, shyness, active circumvention of opportunities for social interaction, and oversensitivity [15]. Children with socially withdrawn behaviour may perceive interpersonal situations as stressful, experiencing social anxiety and thus, they may prefer teacher–child relationships or solitude, reticent behaviours such as being unoccupied, and merely being spectators of social interactions [16]. When they experience peer conflict, they refer to an adult [12,17]. Frequently, they are victimised by their classmates [18].

### 1.2. Aggressive/Withdrawn Behaviours and Externalising/Internalising Symptomatology

Several longitudinal studies have shown that both aggressive and socially withdrawn behaviours in early childhood were related to peer rejection and both predicted externalising and internalising symptoms, respectively, in adolescence (e.g., [18,19,20]). Most researchers agree that aggressive conduct over a school period may cause divergent short- and long-term outcomes [21]. In the short term, children with aggressive behaviour may be found “cool” by a limited range of classmates, but they are commonly isolated and excluded by peers and display poor school performance [3]. In the long-term, while some children tend to engage less in aggressive behaviour by middle childhood and adolescence, others continue to show aggressive behaviour and are at strong risk for later development of chronic antisocial problems (e.g., rule-breaking, delinquency, criminality, and fighting), with serious consequences on social adjustment and academic achievement, and a high likelihood of school drop-out [19,22]. In particular, externalising behaviours constitute the main reason for referring children to mental health services [23]. Moreover, a large body of research seems to confirm that the association between withdrawal and a high level of social anxiety is a strong risk factor for internalising problems, peer rejection and exclusion, and later depression levels [15,24,25].

### 1.3. Teachers’ Reactions to Aggressive and Withdrawn Behaviours

Both aggressive and withdrawn behaviours may represent a challenge for teachers, who have to manage different behavioural patterns and their consequent negative impact on social relationships in the school milieu. Previous studies have already demonstrated that teachers are sensitive to pupils’ aggressive or withdrawn behaviours and hold different attitudes and rules in response to these conducts [26]. Coplan found that preschool teachers perceived children with aggressive behaviours more negatively than those with withdrawn behaviours, and were particularly concerned about the adjustment of children with aggressive or socially withdrawn behaviour who displayed not only willingness to remain alone, but fearful isolation [26]. Deng et al. [27] found that preservice elementary teachers were more likely to show warm feelings to shy children than to exuberant ones. In several studies, researchers used methods in which teachers’ responses were pre-specified in the stimulus material, usually as vignettes about hypothetical pupils [26,27,28]. In this way, teachers did not have the chance to freely express their personal perceptions of aggressive or withdrawn behaviours based on their experience. Additionally, they discussed gender-related differences in general terms, without focusing on each child in the classroom [29].

### 1.4. Aim of the Study

Based on the previous literature review, a clear gap emerges in the current knowledge base, concerning how teachers distinguish pupils’ social conduct and which types of attributions or behavioural aspects drive their descriptions and judgments and the occurrence of social exclusion by peers. Hence, the current study is aimed at filling this gap by exploring how teachers describe, and peers consider, children with aggressive or socially withdrawn behaviours. We used an open-ended semi-structured interview about real children with whom teachers have personal contact, relationship history, and a teaching responsibility towards, and we also collected peers’ nominations, from which we derived the sociometric status of the children in question. Furthermore, given that both aggressive and withdrawn behaviours may contribute to a disruption in peer relationships, it is relevant to take into account the perspectives of classmates.

Specifically, we aimed to answer the following research questions:

1. Do teachers describe children with aggressive behaviour differently than children with socially withdrawn behaviour?
2. Are peers’ nominations of children with aggressive or socially withdrawn behaviours characterised by rejection or neglect? Does the occurrence of rejection or neglect differ according to aggressive or withdrawn behaviours?

## 2. Materials and Methods

### 2.1. Participants

Five teachers used the Child Behaviour Checklist 4–18 Version (CBCL; [7]) to rate all their pupils (*N* = 143; M_age_ = 6.5 years; SD = 1.6; age range = 4–9 years) attending kindergarten and elementary school in central and southern Italy. Subsequently, as suggested by Ohleyer et al. [30], we determined the cut-off for withdrawn and aggressive behaviour to be 1 SD above the mean of the total sample score. Consequently, only children whose scores were 1 SD above the mean for withdrawn behaviour (*n* = 31) or for aggressive behaviour (*n* = 15) were included in the final sample.

### 2.2. Procedure

Permission was obtained from the headteacher of the schools and the institutional schools’ committees, which include teachers and parents. A convenience sampling method was employed: pre-graduate students of Educational Sciences from X University (blind for peer review) who were attending school-based initial teacher training in primary schools involved in the study, and their teacher/mentor (the teacher in charge of the class), who agreed to participate and provided their informed consent, were involved. All procedures performed in this study were conducted in accordance with the ethical standards of the Institutional and National Research Committee and with the 1964 Helsinki Declaration and its later amendments. In addition, all the current study’s activities were conducted in compliance with the rules and regulations set forth by the Ethics Commission of the Department of Education of Roma Tre University. Participation in the study first required written informed consent from parents and teachers, and then verbal consent from children. The teachers in charge of the class, following the procedure recommended by Achenbach [7], individually completed the entire CBCL questionnaire for each child in their class. Then, each teacher was individually interviewed by a trainee teachers about the children that were screened as showing aggressive or withdrawn behaviour in their class.

### 2.3. Measures

Child Behaviour Checklist 4–18 Version (CBCL; [7]): This well-validated instrument consists of 118 problems and 20 competency items, which can be grouped into 11 problem scales and 4 competence scales, or two broader dimensions of internalising and externalising symptoms. In agreement with the object of this study, we were interested in two subscales: withdrawn (Cronbach’s alpha was 0.85 for the school-aged version, and 0.73 for the preschool-aged version in this sample; a cut-off of 6.55 and above was used for the school-aged group, and of 4.03 for the preschool-aged group), and aggressive behaviour (Cronbach’s alpha was 0.90 for the school-aged version, and 0.93 for the preschool-aged version in this sample; a cut-off of 9.65 and above was used for the school-aged version, and of 9.72 for the preschool-aged version). Cut-off scores were determined considering 1 SD above the mean to identify children with withdrawn and aggressive behaviour, respectively, as suggested by Ohleyer et al. [30].

Interview about children’s attributions, behaviours, and emotions [31]: Following Younger et al. [31]’s procedure, for each child in their class who had been screened by the CBCL as a child with aggressive or socially withdrawn behaviour, teachers had to explain how they know said child has aggressive or socially withdrawn behaviours. Once the teacher described a behaviour, he/she was asked to describe the situations in which the behaviour was likely to occur and to describe all the behaviours. Each teacher was individually interviewed by a trainee teacher in an empty classroom of the school. All interviews were audio-recorded and verbatim transcribed

Sociometric Status [32]: The teachers asked their pupils to nominate up to three classmates with whom he/she most likes to be with (like most—LM) and three classmates with whom he/she least likes to be with (like least—LL). Nominations received from all classmates were totalled and standardised within each class. The positive and negative classmate nominations received from peers provided the indexes of social preference (SP = LM − LL) and social impact (SI = LM + LL) which were standardised within each class, and then re-standardised for the entire group of participants. Following the procedure adopted by Slaughter et al. [33], children were classified into one of five peer status groups, although in this study we only focused on popular, rejected, and neglected children.

### 2.4. Data Analyses

In the first part of the Results section, we focused on the teachers’ descriptions, and both qualitative and quantitative data analyses were carried out, also following Braun and Clarke’s suggestions [34]. Specifically, in Step 1, we focused on the transcripts of the teachers’ interviews, becoming familiar with the entire corpus of the data. In Step 2, we organised data, inserted the transcripts for each child in an Excel file, and identified the initial categories, which were organised into broader categories or themes in Step 3. In Step 4, we reviewed themes/categories, verifying possible overlaps among them and considering their sense and relevance. Step 5 encompassed the definition and nomination of themes/categories. Finally, in Step 6, data were reported considering the categories. Furthermore, following a top–down approach, categories in Younger et al. [31] which described peers’ descriptions of children’s social behaviour were considered and compared to the categories that we found. Several additional categories—which were not included in the Younger et al. [31] categorisation—were identified (see Table 1). All transcripts were coded by two independent scorers, whose agreement was very high for the categories of behaviour. Respectively, the two judges had a perfect agreement on the following categories: he/she shows aggression, blushes, avoids eye contact, behaves clumsily, and cries; for the categories untalkative (α = 0.96) and isolated (α = 0.91), the agreement was very high. Regarding attributions, the categories submissive, immature, good relationship with teacher, dominant, and oppositive, had a perfect agreement, whilst the K of hostile was 0.95. The lowest agreement was obtained for passive (α = 0.66) and competitive (α = 0.63) behaviours. There was a perfect agreement for the categories referring to emotions. For the remaining categories, the agreement ranged from 0.80 to 0.89. Any substantial discrepancies between the scorers were resolved by a joint re-examination of the data and consensus coding.

The second part of the Results section was devoted to the relationship between the teachers’ descriptions and peer sociometric status. First, we ran a two-way contingency table (see Table 2) in which the rows represent the teacher-rated CBCL scores, and the columns represent the five sociometric status categories. In particular, we perused the occurrences of children with either aggressive or withdrawn behaviour for each sociometric status. Moreover, we selected and reported the most representative teachers’ descriptions of children with either withdrawn or aggressive behaviour in relation to peer sociometric status.

Second, further descriptions of popular, rejected, and neglected children with either withdrawn or aggressive behaviour were provided, taking into account the occurrences of categories used by teachers to describe the children. In order to make the interpretation of the results easier, categories were grouped into 7 major themes: emotional difficulties, socially retired, oppositional/hostile, compliant, active, competitive, and socially skilled (see Table 3).

## 3. Results

### 3.1. Teachers’ Descriptions

Forty-six children whose scores were 1 SD above the mean for withdrawn or for aggressive behaviour were included in the final sample (M_age_ = 6.5 years; SD_age_ = 1.7; age range = 4–9 years). Among the screened sample, according the teachers, 31 children (21.67%; females = 16) showed withdrawn behaviour, whereas 15 (10.48%; females = 5) showed aggressive behaviour.

From the analysis of the teachers’ interviews, 16 categories of behaviour, 10 categories of attributions, and 2 emotional categories were found. Teachers’ descriptions may encompass more categories for every child. Table 1 enlists these categories and their definitions, and shows the occurrences and the percentages of children with either aggressive or socially withdrawn behaviours.

### 3.2. Aggressive Behaviours and Attributions

Twelve children with aggressive behaviour were described as disruptive and twelve as rule-breaking. They displayed hostile and oppositional attitudes towards other classmates and teachers. One teacher offered the following description of opposition:

*He is an aggressive child because he fights with his classmates and often doesn’t respect adults’ authority and role. One time, during gym hour, he hurt his classmate with a plastic circle. For this action, he received a punishment: He couldn’t play with classmates. However, he continued to display high activity, moving himself excessively in the gym.*

*(Teacher 4)*

### 3.3. Withdrawn Behaviours and Attributions

Conversely, introverted, untalkative, and not participative were the most representative categories for the children with socially withdrawn behaviour. These children talked very little or softly, and they showed low levels of activity. Furthermore, they needed to remain physically close to the teacher. This was referred by their teachers:

*She’s a very introverted child and focused on her own affairs. She doesn’t say anything about herself. She spent the last year alone. Only during recess time, she plays with a girl attending another class. She never smiles in the classroom.*

*(Teacher 1)*

*He doesn’t speak. If I didn’t require something of him, he would remain all day without speaking!*

*(Teacher 2)*

*Since she began school, she has preferred to sit near me in order to interact with me in every moment … She has only one female friend. They spend a lot of time together, not only at school. They attend a modern dance class.*

*(Teacher 2)*

Avoiding eye contact, blushing, and difficulty in coordinating movements were described for 10% of children with withdrawn behaviour, but they were not mentioned for children with aggressive behaviour. Interestingly, both categories of children were described as not involved in social interactions. However, the teachers described different behaviours for two groups of children. In the case of children with withdrawn behaviour, the teacher reported the following:

*She prefers to remain alone… she plays alone. She doesn’t spend time with her classmates.*

*(Teacher 1)*

*She usually plays alone or with only two girls.*

*(Teacher 5)*

For children with aggressive behaviour, the descriptions contained a different motivation for social isolation. One teacher noted the following:

*He’s a very highly active child. He’s often rejected and isolated by his classmates when he hurts or disturbs them.*

*(Teacher 5)*

Regarding emotions, we found that anxiety/fear was repeated in 16 teachers’ descriptions of withdrawn children, whereas they were not used by teachers to depict children with aggressive behaviour. Teachers speculated that children with withdrawn behaviour might fear being wrong and thus receiving negative judgments from peers and teachers. One teacher reported the following:

*If I require him to do something standing in front of his classmates, he rejects my request because he’s afraid of being judged or teased by others.*

*(Teacher 3)*

### 3.4. Peer Nominations and Teachers’ Descriptions

In Table 2, the percentage of peer nominations of children with either aggressive or withdrawn behaviour are shown.

The sociometric status methodology shows that 26.7% of the children with aggressive behaviours and 19.4% of the children with withdrawn behaviour have been rejected. Furthermore, we found that among popular children, only two had withdrawn behaviour (see Table 2). They were described as follows:

*She is shy and insecure. She’s always afraid to make mistakes. In the classroom she is still and tranquil. She becomes red in the face very easily and she often feels anxiety.*

*(Teacher 1)*

*She’s shy, introverted and taciturn. She usually speaks very softly when she must answer a question. For instance, when I read a narrative story in the classroom and I ask questions to pupils, she doesn’t answer aloud, but she whispers because she worries about giving a wrong answer and being teased by her classmates.*

*(Teacher 3)*

Among children with aggressive behaviour, nobody was popular, whereas four were classified as rejected and two as neglected. The descriptions provided by the teachers and the peer nominations show some similarities. Both rejected and neglected children were described as aggressive, rule-breaking, active, oppositional, and hostile. Furthermore, neglected children did not receive any teachers’ description regarding the socially skilled category (see Table 3).

*She is an aggressive child. If another person doesn’t agree with her, she displays an aggressive conduct. For instance, when her mate doesn’t do what she desires, she seeks revenge, breaking the mate’s toys, saying unpleasant words, or hurting him/her. She is also very jealous, and she is very aggressive if a child takes her things.*

*(Teacher 3)*

*He’s often happy and active. He plays heavy physically impacted games with his mates. However, he engages in controversial interactions with peers. His classmates seek his participation during games, but they reject him when he becomes excessively active. He disturbs his mates and thus, they isolate him. When this occurs, he engages in immature and oppositional attitudes. He damages others’ toys or steals things which are indispensable to complete a game or an activity. Moreover, he often disturbs the lesson. He hurts his classmates or teases them. He provokes us (the teachers) and avoids respecting rules and requests.*

*(Teacher 5)*

The first description refers to a child with aggressive behaviour who received very high negative nominations, whereas the second one depicts a neglected child. In the latter case, the aspects of immaturity and opposition are very clear, and the classmates do not reject, but ignore him.

Among the children with socially withdrawn behaviour, six were rejected and five were neglected. Both groups preferred to play alone and were depicted by their teachers as socially retired and with emotional difficulties. Specifically, the categories clumsy and compliant, and participation in passive games were only used for withdrawn, neglected children, as is evident in the following description:

*He prefers playing with few mates. It is very difficult for him to talk about his feelings and emotions and ask for help. He’s often clumsy and messy. He displays difficulty when he must speak to strangers and even when he speaks with familiar people, he’s very introverted and shy. He never begins a game with peers spontaneously. When he’s involved by mates, he plays with them, but always assuming a marginal role and displaying fear to receive a negative judgment. He complains and prefers playing with friends who like passive games and use polite attitudes.*

*(Teachers 5)*

## 4. Discussion

### 4.1. Children with Aggressive Behaviour: Teachers’ Descriptions and Peer Sociometric Status

The aim of this study was to combine the teachers’ descriptions and peer sociometric status of children with socially withdrawn or aggressive behaviours. Regarding children with aggressive behaviour, Arbeau and Coplan [28] and Coplan et al. [26] found that teachers expressed harsher beliefs toward aggressive conduct compared to unsociable behaviour. In our study, behaviours of opposition to the teacher were described. These behaviours could imply some sort of agonistic relationship between these children and the teacher, which could easily elicit anger in the teachers. Children were described as engaging in a direct form of aggression more than in an indirect (relational) one. This result is consistent with past studies showing how advanced indirect forms of peer aggression, such as disseminating malicious rumours among classmates, are uncommon experiences among young children; the only relational forms which may be present in very young children are very simple, and are usually overt and direct, such as turning the face away in order to ignore a classmate [35,36].

Moreover, children with aggressive behaviour, either rejected or neglected by their peers, were depicted by teachers as oppositional and hostile; in addition, no social skills were reported by teachers when describing neglected children. Our results are in line with evidence from cross-sectional and longitudinal studies confirming a strong relation between peer rejection and different forms of aggressive behaviours in childhood and in adolescence (for a meta-analysis, see [5]). These social dynamics may partially explain the link between the direct forms of aggression in childhood and future externalising conduct (e.g., [18,19,20]).

It is also very interesting to note that a minority of rejected children with aggressive behaviour were rated as socially competent. This subgroup of socially skilled aggressive children has been discussed in the literature [37]. Previous studies [38,39] have suggested that children with aggressive behaviour appear to possess socio-cognitive abilities, but they tend to use them for antisocial purposes.

### 4.2. Children with Withdrawn Behaviour: Teachers’ Descriptions and Peer Sociometric Status

Regarding children with withdrawn behaviour, the teachers’ descriptions in our study are very similar to peers’ descriptions in the study by Younger et al. [28], but with different percentages, showing that adults and peers focus on different aspects. While in our study the most used category by teachers was “doesn’t participate”, followed by introverted, untalkative, frightened, and close to teachers, in the study by Younger et al. [28] the most used category by peers was “doesn’t talk”, followed by stays by self, does not play/participate, and runs/walks away from others. The description of children with withdrawn behaviour as untalkative allows us to focus attention on the role of relevant social competence, such as communicative competence which has been found to be associated with sociometric status [40]. Furthermore, teachers took into account the tendency of children with withdrawn behaviour to be on their own, even though in both studies, very few of them were described as avoiding eye contact with others. Teachers, differently from peers, used more psychological terms such as “introverted”, and recognised signs of anxiety or distress in children with withdrawn behaviours, whereas no reference to the emotional realm was mentioned in the descriptions provided by peers. In our study, children with withdrawn behaviours who had been described by teachers as socially retired (e.g., standing by themselves) were classified as either rejected or neglected by their peers. This association is well known in the literature [17]. Many researchers (e.g., [31,41]) have suggested paying attention to how classmates interpret the motivation behind behaviours of children with socially withdrawn behaviour when staying by themselves. It is reasonable to think that peers tend to exclude or neglect classmates whose solitary behaviour is evaluated as a sign of disinterest or unfriendliness.

Most of the teachers’ descriptions of children with withdrawn behaviour are also consistent with the construct of unsociability. The latter refers to an unfearful preference for simply being alone [42], whereas fear, anxiety, and wariness in the face of situational demand for social interaction are more easily associated with withdrawal and shyness. In the attempt to concretely differentiate these concepts, Rubin et al. [43], Gazzelle and Ladd [24], and Ladd et al. [44] have distinguished anxious-solitary children from unsociable children. A large body of research has broadly documented that fearful withdrawal/shyness is a greater risk factor for internalising problems, peer rejection, and depression than unsociability [43]. Our study seems to support the distinction between unsociability and fearful withdrawal/shyness. Our teachers used fear and anxiety terms for only half of the descriptions of children with withdrawn behaviours.

### 4.3. Peer Rejection and Isolation

Most of the children with socially withdrawn behaviour and a third of children with aggressive behaviour were described as staying by themselves. Moreno [45], the pioneer of peer relations research, believed that remaining on one’s own is an observable marker of peer rejection. Nevertheless, our data show that children’s isolation observed by teachers does not necessarily coincide with sociometric rejection; for our children with withdrawn behaviours, in some cases, it could be a choice. Consequently, based on our results and following Coplan et al. [41]’s considerations, from an observational point of view, we may distinguish three types of isolation: children who stay alone because they are socially fearful, those who prefer to stay alone, and those who stay alone because they are rejected. The teacher’s intervention should take into account these differentiations, specifically focusing on the fear, the willingness of being alone, or the peer rejection.

### 4.4. Limits and Future Research Directions

The combined use of peer nominations and teachers’ descriptions represents the main advantage of the current study, whose results add to the existing literature dealing with social conduct and sociometric status in the educational context. However, caution is required in interpreting the findings of the study because of a few limitations. Firstly, the low sample size did not allow us to generalise on a population, nor to compute parametrical analyses. These observations may suggest future directions of research both in increasing the sample size and exploring data not only from teachers but also from families to assess variability.

Secondly, no information on the stability of aggressive or withdrawn behaviour across childhood was included due to the cross-sectional nature of the present study. Thirdly, more measures could be employed to assess socio-emotional skills. Finally, sociometric status was derived from peer nominations. Although these issues are fundamental to peer relations research, more structured observational data would complement the data in this field of study.

## 5. Conclusions

Despite teachers being encouraged to describe pupils’ behaviours, some of them proceeded directly to attributional statements, seemingly without attending to the behaviours associated with the attributions. This attitude is of some concern, especially when those attributions and labels were not grounded on the basis of actual aggressive behaviours. Teachers’ attributions of hostility, if not grounded on actual behaviours, may contribute to shaping a child’s image as aggressive and consequently reinforce his/her aggressive tendencies [12]. It is important to train future teachers to observe the peculiarity of their pupils and to ground their descriptions on actual behaviours, as opposed to judgements, prejudices, labels, and stereotypes. Regarding children’s feelings, perceptions and behaviours, Sommer et al. [46] distinguished a child perspective (adults’ perspective on children) from a child’s perspective (child’s internal perspective on what he or she finds as meaningful). Teachers, parents, and health professionals should include the child’s perspective in their child perspective, and direct their efforts to being attentive, sensitive, and supportive of each child’s expressions, experiences, and perceptions of interpersonal relationships [47]. The improvement of this particular teacher’s ability could ensure not only a better teacher–student relationship, even when the latter has some issues with socialisation, but also, when more serious child behavioural problems are present, their early identification could be useful to plan intervention programs.

In summary, the adjectives introverted and untalkative have been adopted by teachers when describing children with socially withdrawn behaviours, whereas hostile, rule-breaking, and highly active were terms used when depicting children with aggressive behaviour. When sociometric status was considered, most children with socially withdrawn behaviour and those with aggressive behaviour were classified as rejected or neglected.

Repetitive experiences of peer rejection and peer neglect in childhood may contribute to a limitation in the development and practice of social skills, which are essential for peer social interactions [48]. Moreover, early poor peer relations may exacerbate the risk for social isolation or exclusion from formal peer groups in adolescence, a developmental phase in which peer pressure and judgment are very strong, as well as the susceptibility to mental health problems [49]. Hence, early recognition of signs underpinning peer relation difficulties is crucial, and specific training targeting social and emotional competencies can effectively contribute to reducing aggressive [50] and withdrawn behaviours [51].

In line with the findings of both the present study and previous research, it is recommended that, rather than labelling these children or referring them to mental health services, teachers and educators should be provided with with specific training programs targeting observation skills. Such programs should (a) encourage them to focus attention on and describe children’s behaviour while avoiding unfounded attributions, and (b) recognise withdrawn or aggressive behaviours early and identify other social difficulties before they cause peer rejection and neglect. On the other hand, it is essential to provide educators and teachers with school-based programs aimed at promoting children’s socio-cognitive abilities and emotional competencies. Finally, from a research point of view, longitudinal studies are needed to further understand the protective and risk factors underpinning withdrawn and aggressive behaviour in childhood.

## Figures and Tables

**Table 1:** Frequencies of behaviours, attributions, and emotions mentioned by teachers.

Category	Definition	Raw Frequency Socially Withdrawn *n* = 31 (%)	Raw Frequency Aggressive *n* = 15 (%)
*Behaviours*			
Aggressive/Disruptive	Child physically hurts another person	0	**12 (80)**
Rule-breaking/Oppositional	Behaviour inconsistent with school rules and teacher’s instructions	4 (12.9)	**12 (80)**
High Activity	Child moves around excessively	0	**5 (33.3)**
Easily Distressed	Behaviours indicating distress or anxiety	6 (19.4)	2 (13.3)
Blushes or Nervous movements	Movement indicative of anxiety, such as scratching head	5 (16.1)	0
Clumsy	Poorly coordinated physically	3 (9.7)	0
Cries		4 (12.9)	2 (13.3)
Avoids eye contact	Does not look at others directly, lowers head	3 (9.7)	0
Inattentive	Unfocused, attention wanders, disoriented	4 (12.9)	1 (6.7)
Does not participate or rejected	Stays by self, plays alone, does not participate, or rejected by others	*23 (74.2)*	***5 (33.3)***
Untalkative	Talks very little, or with low voice volume	*20 (64.5)*	1 (6.7)
Passive games or activities	Chooses games or activities with low movement or noise	9 (29.0)	0
Physical closeness to teacher	Remains physically close to teacher	*10 (32.3)*	0
Helpful, Generous	Helps others	4 (12.9)	1 (6.7)
Competitive	Child leads or wants to win	0	4 (26.7)
Compliant	Obeys teacher	3 (9.7)	1 (6.7)
*Attributions*			
Hostile	Intends to harm another person	1 (3.2)	**12 (80.0)**
Oppositional	Irreverent attitude towards adult authority	1 (3.2)	**7 (46.7)**
Introverted	Quiet and unable to make friends easily	*22 (71.0)*	1 (6.7)
Distressed/Insecure	Upset or worried	*7 (22.6)*	2 (13.3)
Immature	Seems like a younger child	1 (3.2)	3 (20.0)
Good relationship with teacher		0	2 (13.3)
Socially Skilled	Has competency or disposition to initiate and maintain relationships with others	*5 (16.1)*	3 (20.0)
Submissive	Defers to others’ wishes	4 (12.9)	0
Dominant	Wants to win or lead games or situations	1 (3.2)	2 (13.3)
Active	Lively by temperament	3 (9.7)	**5 (33.3)**
*Emotions*			
Fear/Anxiety		17 (51.5)	0
Other negative emotions	Sadness, embarrassment, jealousy, frustration	3 (9.1)	2 (12.5)

*Note.* Categories indicated in boldface are the three most frequent for aggressive children in each section (behaviours or attributions). Categories in italics are the three most frequent for withdrawn children. Categories in both bold and italics are common among both socially withdrawn and aggressive children.

**Table 2:** Peer sociometric status of children with aggressive and withdrawn behaviour.

Status	Raw Frequency (%)	
	Aggressive Behaviour	Withdrawn Behaviour
Popular	0 (0.0)	2 (6.5)
Rejected	**4 (26.7)**	**6 (19.4)**
Neglected	2 (13.3)	**5 (16.1)**
Controversial	**2 (13.3)**	1 (3.2)
Average	**7 (46.7)**	**17 (54.8)**
Total	15 (100.0)	31 (100.0)

*Note*. Categories indicated in boldface are the three most frequent.

**Table 3:** Peer sociometric status and teachers’ descriptions of children with aggressive or withdrawn behaviour.

	Behaviour																		
Peer Sociometric Status	Aggressive						Withdrawn												
	Rejected				Neglected		Rejected						Neglected					Popular	
	C_1_	C_2_	C_3_	C_4_	C_5_	C_6_	C_7_	C_8_	C_9_	C_10_	C_11_	C_12_	C_13_	C_14_	C_15_	C_16_	C_17_	C_18_	C_19_
*Teachers’ Descriptions*																			
Emotional difficulties	2					1	1	1		1		1	4			1	1	3	
Socially retired	1			1		1	1	3	2	2	3	3	2	2	3	1	4		3
Oppositional/hostile	3	2	3	4	4	3												1	
Compliant							1	1			2	3	2	1		2	1		
Active			2	1		2													
Competitive				2									1						
Socially skilled	1			2			1	1			1	2							

*Note.* C = child. Categories are summed up as follows, including behaviours, attributions, and emotions. Emotional difficulties = easily distressed, blushes or nervous movements, distressed/insecure, cries, clumsy, immature, fear/anxiety, and other negative emotions; socially retired = inattentive, does not participate or rejected, avoids eye contact, untalkative, inattentive, and introverted; complaint = submissive, compliant, and passive games or activities; oppositional/hostile = hostile, oppositional, aggressive/disruptive, and rule-breaking/oppositional; active = high activity and active; competitive = competitive and dominant; socially skilled = good relationship with teacher, helpful, generous, and socially skilled.

## Data Availability

The data presented in this study are available on request from the corresponding author. The datasets generated and/or analysed during the state study are not publicly available due to privacy, confidentiality, and other restrictions.
